# Bacteria-Catalyzed Arginine Glycosylation in Pathogens and Host

**DOI:** 10.3389/fcimb.2020.00185

**Published:** 2020-04-28

**Authors:** Xing Pan, Jie Luo, Shan Li

**Affiliations:** ^1^Institute of Infection and Immunity, Taihe Hospital, Hubei University of Medicine, Shiyan, China; ^2^College of Biomedicine and Health, Huazhong Agricultural University, Wuhan, China; ^3^College of Life Science and Technology, Huazhong Agricultural University, Wuhan, China

**Keywords:** arginine glycosylation, T3SS effectors, death receptor signaling, NleB, SseK, EarP, posttranslational modification, glycosyltransferase

## Abstract

In recent years, protein glycosylation in pathogenic bacteria has attracted more and more attention, and accumulating evidence indicated that this type of posttranslational modification is involved in many physiological processes. The NleB from several enteropathogenic bacteria species as well as SseK from *Salmonella enterica* are type III secretion system effectors, which have an atypical N-acetylglucosamine (N-GlcNAc) transferase activity that specifically modified a conserved arginine in TRADD, FADD, and RIPK1. NleB/SseKs GlcNAcylation of death domain proteins abrogates homotypic and heterotypic death receptors/adaptors interactions, thereby blocking an important antimicrobial host response. Interestingly, NleB/SseKs could also GlcNAcylate themselves, and self-GlcNAcylation of NleB, SseK1, and SseK3 are crucial for their biological activity during infection. In addition, EarP (EF-P specific arginine rhamnosyl transferase for Posttranslational activation) catalyzes arginine rhamnosylation of translation elongation factor P (EF-P). Importantly, this kind of N-linked protein glycosylation is not only important for EF-P dependent rescue of polyproline stalled ribosomes but also for pathogenicity in *Pseudomonas aeruginosa* and other clinically relevant bacteria. Glycosylation of arginine is unique because the guanidine group of arginine has a high acid dissociation constant value and representing an extremely poor nucleophile. Recently, the crystal structures of NleB, SseKs, EarP, arginine GlcNAcylated death domain-containing proteins, NleB/FADD-DD, and EarP/EF-P/dTDP-β-L-rhamnose were solved by our group and other groups, revealing the unique catalytic mechanisms. In this review, we provide detailed information about the currently known arginine glycosyltransferases and their potential catalytic mechanisms.

## Introduction

Glycosylation is one of the most abundant and complex posttranslational modifications of proteins and involved in diverse processes such as cell differentiation and growth, signaling cascades, tumorigenesis, as well as host-pathogen interactions (Haltiwanger and Lowe, 2004; Marth and Grewal, 2008; Lu et al., 2015; Stowell et al., 2015). Additionally, glycosylation affects protein properties including stability, folding, and solubility (Moremen et al., 2012). Glycosyltransferases (GTs) are enzymes that establish natural glycosidic linkages, which catalyze the transfer of a sugar moiety from a glycosyl donor to a substrate, and are classified on the basis of their structure as GT-A, GT-B, GT-C, or GT-D fold (Lairson et al., 2008; Gloster, 2014; Zhang et al., 2014; Liang et al., 2015; Park et al., 2018). The GT-A type GT is composed of two β/α/β Rossmann domains, and the two domains are closely interlinked. Out of this, GT-A enzymes possess a conserved Asp-X-Asp (X is any amino acid) motif in which the carboxylates coordinate Mn^2+^/Mg^2+^. Just as the GT-A type, the structure of GT-B enzymes is made up of two β/α/β Rossmann-like domains as well. However, in contrast with the architecture of GT-A enzymes, the two β/α/β Rossmann domains in GT-B are linked flexibly. In addition, the metal ions are not required in GT-B enzymes either. The GT-C fold enzymes have numbers of transmembrane helices, and they also have a long-loop region within an active site. One study showed that DUF1792 has a Rossmann-like fold, but the sequence and structure are quite different with the currently annotated type GT-A, GT-B, or GT-C folds, which is why it was defined as a GT-D glycosyltransferase fold.

According to the glycosidic linkage, protein glycosylation can also be divided into O-linked or N-linked glycosylation (Jensen et al., 2012; Rojas-Macias et al., 2019). Typically, O-linked glycans are conjugated to the hydroxyl oxygen of serine and threonine (Van Den Steen et al., 1998; Yang and Qian, 2017). In contrast, N-linked glycans are usually linked to the amide side chain of an asparagine residue in an N-X-S/T (X can be any amino acid, but not proline) motif (Shakin-Eshleman et al., 1996; Mellquist et al., 1998). Unlike with the popular and fully understood N-linked glycosylation of asparagine, N-acetylglucosaminylation (N-GlcNAcylation) that occurs on arginine is quite rare, and the special molecular mechanism of arginine N-GlcNAcylation is completely unknown. It has so far only three reported cases of arginine glycosylation. Self-β-arginine glycosylation was discovered first in sweet corn protein amylogenin in 1995 (Singh et al., 1995). However, there have been no follow up reports. Secondly, the NleB from several enteropathogenic bacteria species as well as SseK from *Salmonella enterica* are type III secretion system (T3SS) effectors, which were shown to inactivate host death receptors/adaptors by an unprecedented N-GlcNAcylation of a conserved arginine (Li et al., 2013; Pearson et al., 2013). NleB/SseK manipulate host death receptor signaling pathways facilitate the pathogens infection and evade host immune defenses. NleB homologs are present in pathogenic *Escherichia coli, Citrobacter rodentium* (NleBc), and *S. enterica* (SseK1/2/3) (Deng et al., 2004; Araujo-Garrido et al., 2020). It should be noticed that enteropathogenic and enterohemorrhagic *E. coli* (EPEC and EHEC) have two copies of NleB, termed NleB1 and NleB2, and share about 61% amino acid sequence homology (Perna et al., 2001; Iguchi et al., 2009). In addition, EPEC NleB1, EPEC NleB2, EHEC NleB1, EHEC NleB2, SseK1, SseK2, and SseK3 is about 89, 60, 89, 60, 57, 53, and 52% identical to *C. rodentium* NleB, respectively (Araujo-Garrido et al., 2020). Interestingly, when compared with NleB1/NleBc/SseK1/3, NleB2, and SseK2 possessed a much lower GlcNAcylation activity (Li et al., 2013; Pearson et al., 2013; El Qaidi et al., 2017; Gunster et al., 2017; Newson et al., 2019). In the third case, a conserved arginine of the bacterial translation elongation factor P (EF-P) is rhamnosylated by EarP (EF-P specific arginine rhamnosyl transferase for Posttranslational activation) (Lassak et al., 2015; Rajkovic et al., 2015; Yanagisawa et al., 2016). Notably, this unique modification is important for EF-P dependent rescue of polyproline stalled ribosomes in clinically relevant bacteria such as *Pseudomonas aeruginosa* and *Neisseria meningitides* (Lassak et al., 2015; Yanagisawa et al., 2016). Moreover, several studies have shown that EF-P and EarP contribute to the pathogenicity of *P. aeruginosa* and *N. meningitidis* by controlling the translation of proline stretch-containing proteins critical for modulating motility, antibiotic resistance, and other traits that play key roles in establishing virulence (Lassak et al., 2015; Rajkovic et al., 2015; Yanagisawa et al., 2016).

Here we provide a summary of bacterial arginine glycosyltransferases and their targets in recent research progress, the unique catalytic mechanisms for arginine glycosylation are discussed as well.

## Arginine N-Acetylglucosamine Transferase in Pathogenic *E. COLI* and *C. Rodentium*

EPEC is an attaching/effacing (A/E) pathogen that usually leads to severe watery diarrhea, which remains a serious health issue in developing countries (Kotloff et al., 2013). A related *E. coli* pathotype, EHEC, is the predominant pathogen of bloody diarrhea and hemolytic uremic syndrome (HUS) (Nguyen and Sperandio, 2012). These human bacterial pathogens, together with *C. rodentium*, a natural murine intestinal bacterium that behaved as the related human pathogens EPEC and EHEC, usually translocate a core set of effectors into host cells to antagonize host defense (Gaytan et al., 2016; Pearson et al., 2016; Pinaud et al., 2018; Shenoy et al., 2018).

Pathogenic *E. coli* and *C. rodentium* T3SS effectors, such as Tir (Ruchaud-Sparagano et al., 2011), EspL (Pearson et al., 2017), NleB (Nadler et al., 2010; Newton et al., 2010), NleC (Yen et al., 2010; Baruch et al., 2011; Muhlen et al., 2011; Pearson et al., 2011; Shames et al., 2011; Sham et al., 2011), NleD (Baruch et al., 2011; Creuzburg et al., 2017), and NleE (Nadler et al., 2010; Newton et al., 2010; Zhang et al., 2011), all of which could manipulate the host innate immune system, including the nuclear factor kappa-light-chain-enhancer of activated B cells (NF-κB) signaling and death receptor signaling, via several different mechanisms. It should be noticed that NleB is required for virulence of *C. rodentium in vivo* (Kelly et al., 2006; Wickham et al., 2006). More importantly, several studies suggested that NleB, to some extent, is associated with the prevalence of human EHEC outbreaks and the outcome of infection (Wickham et al., 2006).

**Figure 1:**
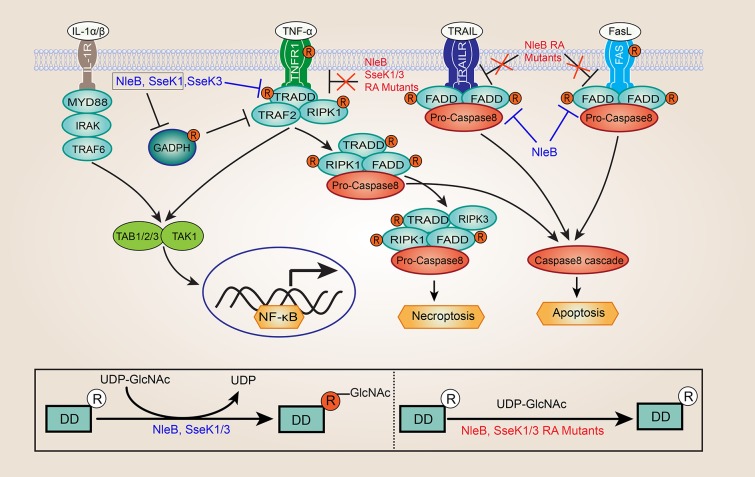
Inhibition of NF-κB signaling and death receptor signaling by NleB and SseK1/3. GlcNAcylation of GAPDH by NleB/SseK1 would suppress TRAF2 polyubiquitination and NF-κB activation. GlcNAcylation of death domain (DD) proteins by NleB and SseK1/3 abrogates homotypic and heterotypic death receptors/adaptors interactions and the assembly of TNFR1 complex, leading to disrupting TNF signaling in EPEC or *Citrobacter rodentium* infected cells, including NF-κB signaling, apoptosis, and necroptosis. NleB also blocked Fas ligand and TNF-associated apoptosis-inducing ligand (TRAIL)-induced cell death by preventing assembly of the canonical death inducing signaling complex (DISC). In contrast, interleukin-1 receptor-associated kinase1 (IRAK1), and myeloid differentiation primary response 88 (MYD88), lacking the conserved arginine were not GlcNAcylated by NleB and SseK1/3. Besides, the site-directed RA mutants of NleB (NleB_Arg13/53/159/293Ala_), SseK1 (SseK1_Arg30/158/339Ala_), and SseK3 (SseK3_Arg153/184/305/335Ala_) abolished or attenuated the capability of enzyme activity toward their death domain-containing targets during infection, and loss of self-GlcNAcylation of NleB, SseK1, and SseK3 couldn't inhibit TNFα- or TRAIL-induced cell death.

In 2010, Nadler et. al and Newton et. al reported that both NleE and NleB could inhibit NF-κB activation (Nadler et al., 2010; Newton et al., 2010). However, the inhibition activity of NleE and NleB is different, NleE could inhibit both TNFα and IL-1β stimulated NF-κB activation, whereas NleB effector could only inhibit the TNF signaling pathway (Newton et al., 2010; Ruchaud-Sparagano et al., 2011). Although it is well-known that NleB plays an important role in the suppresses NF-κB activation, but the underlying mechanisms are poorly understood. In 2013, one study proposed that glyceraldehyde 3-phosphate dehydrogenase (GAPDH) was the target of *C. rodentium* NleB, and NleB acted as an O-GlcNAc transferase that modified GAPDH (Gao et al., 2013). Glycosylation of GAPDH inhibited the activity of the tumor necrosis factor receptor-associated factors 2 (TRAF2) and TRAF3, thereby leading to reduced NF-κB signaling and type I IFN signaling (Gao et al., 2013, 2016). Just a few months later, two research groups discovered independently that a critical function of NleB in hijacking of the host death receptor signaling and interfering with host defense (Li et al., 2013; Pearson et al., 2013). Surprisingly, NleB has an unusual N-GlcNAcylation activity toward a conserved arginine (Arg235 in TRADD, Arg117 in FADD, and Arg603 in RIPK1) in host death domain-containing proteins (Li et al., 2013; Pearson et al., 2013). NleB GlcNAcylation of these proteins abrogated homotypic and heterotypic death receptors/adaptors interactions, resulting in disrupting TNF signaling in EPEC or *C. rodentium* infected cells (Figure 1) (Li et al., 2013; Pearson et al., 2013; Ding et al., 2019). NleB could also block Fas ligand and TNF-associated apoptosis-inducing ligand (TRAIL)-induced cell death by preventing assembly of the canonical death inducing signaling complex (DISC) (Figure 1) (Li et al., 2013; Pearson et al., 2013; Ding et al., 2019). It should be noticed that other host death domain-containing adaptors, such as interleukin-1 receptor-associated kinase1 (IRAK1) and myeloid differentiation primary response 88 (MYD88), located downstream of the of IL-1 receptor (IL-1R), and lack the conserved arginine were not GlcNAcylated by NleB (Figure 1) (Li et al., 2013). This finding perfectly explained the previous observation that NleB could selectively inhibit TNF-α but not IL-1β activation of the NF-κB signaling (Newton et al., 2010; Ruchaud-Sparagano et al., 2011). Further, hijacking of host death receptor signaling pathways by NleB was required for *C. rodentium* colonization in the mouse model (Li et al., 2013; Pearson et al., 2013; Wong Fok Lung et al., 2016). In addition, several studies suggested that GAPDH is another specific target of EHEC NleB1, and EHEC NleB1-mediated GAPDH GlcNAcylation at Arg197 and Arg200 (Gao et al., 2013; El Qaidi et al., 2017, 2018; Park et al., 2018).

Hypoxia-inducible factor 1-alpha (HIF-1α), which act as a key regulator of cellular O_2_ homeostasis, plays an important role in regulating oxidative glucose metabolism and glycolytic gene expression in the glucose metabolism pathway (Iyer et al., 1998; Cheng et al., 2014; Shukla et al., 2017). Remarkably, in more recent work, Wuhan Xiao and co-workers discovered that HIF-1α was GlcNAcylated at a conserved arginine (Arg18) during *C. rodentium* or EPEC infection, and the modification enhanced HIF-1α transcriptional activity, thus inducing downstream glucose metabolism-associated gene [such as glucose transporter 1 (*GLUT1*) gene] expression to alter host glucose metabolism (Xu et al., 2018). Additionally, a more recently study has shown that bacterial glutathione synthetase (GshB) was GlcNAcylated by *C. rodentium* NleB on Arg256 (El Qaidi et al., 2020). Further, NleB-mediated GlcNAcylation of GshB contributed to *C. rodentium* survival in oxidative stress conditions (El Qaidi et al., 2020).

## Arginine N-Acetylglucosamine Transferase in *S. Enterica*

*S. enterica* is a motile, non-spore-forming, and intracellular Gram-negative pathogen that causes both localized and systemic diseases in a wide range of mammals (Gal-Mor et al., 2014). Unlike EPEC, which possesses one T3SS, pathogenic serovars of *Salmonella* possesses two T3SSs, T3SS1 (encoded by *Salmonella* pathogenicity islands 1, SPI-1), and T3SS2 (encoded by SPI-2), that inject numerous of effectors into host cells to benefit bacterial invasion and survival (Pearson et al., 2016; Pinaud et al., 2018). Interestingly, SPI-1 and SPI-2, which are activated at different infection stages. T3SS1 is activated upon contact with intestinal epithelial cells, and responsible for the invasion (Zhang et al., 2018; Lou et al., 2019). In contrast, the T3SS2 is expressed after *Salmonella* has entered host cell (Figueira et al., 2013; Jennings et al., 2017). Moreover, T3SS2 effectors manipulate vesicular trafficking, thereby enhancing *Salmonella* intracellular survival (Jennings et al., 2017). Three closely related *Salmonella* T3SS effectors, SseK1, SseK2, and SseK3, are translocated by the SPI-2 T3SS, behave as NleB-like arginine glycosyltransferase, although they displayed distinct differences in host substrate specificity (Brown et al., 2011; Li et al., 2013; Pearson et al., 2013; El Qaidi et al., 2017). Data by Günster et. al suggested SseK1 caused the GlcNAcylation of TRADD and FADD, whereas SseK3 resulted in weak GlcNAcylation of TRADD but not FADD (Gunster et al., 2017). However, another study reported that SseK1 only glycosylated GAPDH but not FADD *in vitro* (El Qaidi et al., 2017). Outside of this, a recent report coupled with our study provided evidence that endogenous SseK1 modified TRADD, but not FADD, and endogenous SseK3 modified TNFR1 (at Arg376) and TRAILR (at Arg293) during *Salmonella* infection (Newson et al., 2019; Pan et al., 2019). Significantly, in line with the biological activity of NleB effector, SseK1 and SseK3 inhibit NF-κB activity as well as TNFα-induced cell death (Figure 1) (Gunster et al., 2017; Newson et al., 2019). In contrast with SseK1 and SseK3, SseK2 result in inhibition of TNFα-induced NF-κB reporter activation *in vitro* only (Gunster et al., 2017; Newson et al., 2019). Additionally, SseK2 didn't induce detectable arginine GlcNAcylation during *Salmonella* (ΔsseK1/3 or ΔsseK1/3+pSseK2) infection (Gunster et al., 2017; Newson et al., 2019). Therefore, SseK2 may have a much weaker enzyme-substrate interaction network when compared with SseK1 and SseK3. Remarkably, TRIM32, an E3 ubiquitin ligase, was the first potential binding partner identified for SseK3 (Yang et al., 2015). However, SseK3 could not glycosylate TRIM32. Besides, TRIM32 was not required for SseK3 to inhibit NF-κB signaling either (Yang et al., 2015).

Interestingly, two studies have noticed that in addition to GlcNAcylation of TRADD, FADD, and RIPK1, NleB/SseKs could also GlcNAcylate themselves when over-expressed, though the functional significance of this modification is almost completely unknown (Park et al., 2018; Newson et al., 2019). Fortunately, in a more recent study, our study revealed that Arg13/53/159/293 in NleB, Arg30/158/339 in SseK1, and Arg153/184/305/335 in SseK3 were the self-GlcNAcylation sites, which is consistent with the results from one previous study (Newson et al., 2019; Pan et al., 2019). Moreover, the site-directed mutants, i.e., NleB_Arg13/53/159/293Ala_ (NleB RA mutant), SseK1_Arg30/158/339Ala_ (SseK1 RA mutant), and SseK3_Arg153/184/305/335Ala_ (SseK3 RA mutant), abolished or attenuated the enzyme activity toward their death domain-containing targets (Figure 1) (Pan et al., 2019). Importantly, the NleB RA mutant, the SseK1 RA mutant, and the SseK3 RA mutant could not inhibit TNFα- or TRAIL-induced cell death (Pan et al., 2019).

## Arginine Rhamnosyltransferase Earp of *P. Aeruginosa* and Other Clinically Relevant Bacterial Species

Ribosomes are the workplaces of protein biosynthesis, the process of translating mRNA into protein. Surprisingly, the ribosomes form peptide bonds among different amino acids with various efficiency. Proline is the least efficient one, both as a donor in the peptidyl-tRNA binding site (P-site) and as an acceptor in the tRNA exiting site (E-site) (Muto and Ito, 2008; Pavlov et al., 2009; Johansson et al., 2011). In this case, ribosomes stall when met with an XPP/PPX motif (Elgamal et al., 2014). Ribosome stalling at polyproline motifs is rescued by the eukaryotic and archaeal elongation factor 5A (e/aIF5A) and its bacterial ortholog, the EF-P, but only when e/aIF5A and EF-P is post-translationally activated (Saini et al., 2009; Zou et al., 2012; Gutierrez et al., 2013; Hersch et al., 2013; Peil et al., 2013; Ude et al., 2013). In bacteria such as *E. coli*, EF-P is activated when the conserved Lys34 (K34 is conserved in the EF-Ps among about 80% of bacteria) is (*R*)-β-lysinylated and hydroxylated (Zou et al., 2012; Hersch et al., 2013; Peil et al., 2013; Ude et al., 2013). Comparably, in eukaryotes, conserved Lys50 in e/aIF5A is extended to hypusine (Saini et al., 2009; Gutierrez et al., 2013).

**Figure 2:**
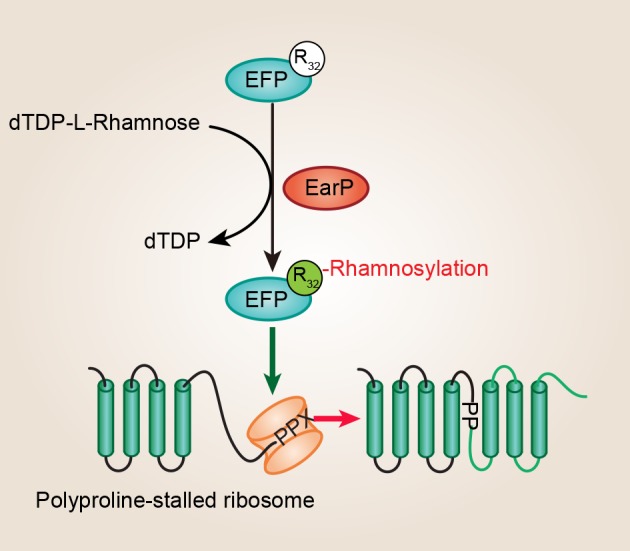
EF-P arginine rhamnosylation and mode of action. A conserved arginine (Arg32) of EF-P is rhamnosylated by EarP using dTDP-β-L-rhamnose as a sugar donor, and this type of posttranslational modification is crucial for EF-P dependent rescue of polyproline stalled ribosomes. EF-P, translation elongation factor P; EarP, EF-P specific arginine rhamnosyl transferase for posttranslational activation.

Unusually, several recent studies discovered that the EF-P (*efp* and *earP* are always directly adjacent) proteins from about 9% of bacteria, such as *Shewanella oneidensis, P. aeruginosa, N. meningitidis, N. gonorrhoeae*, and *Bordetella pertussis*, have a conserved Arg at the position corresponding to Lys34 (Lassak et al., 2015; Rajkovic et al., 2015; Yanagisawa et al., 2016). Significantly, Arg32 in EF-P is rhamnosylated by EarP, and this type of posttranslational modification strategy is crucial for EF-P dependent rescue of polyproline stalled ribosomes in *P. aeruginosa* and *N. meningitidis* (Figure 2) (Lassak et al., 2015; Rajkovic et al., 2015; Yanagisawa et al., 2016). *P. aeruginosa* is a Gram-negative, rod-shaped, asporogenous, and monoflagellated bacterium, and is classified as an opportunistic pathogen (De Lorenzo, 2015). It has been reported that lots of *P. aeruginosa* virulence factors, such as rhamnolipids and pyocyanin, are polyproline-containing proteins, showing a dependence on EF-P for their translation (Lassak et al., 2015). Moreover, proteins involved with motility, protein synthesis, and DNA replication also act as putative EF-P targets in *P. aeruginosa* (Rajkovic et al., 2015). Therefore, EF-P and EarP are crucial for *P. aeruginosa* pathogenicity. In total, this is the first example of N-linked glycosylation occurring on arginine in bacteria which is important for its own biological function.

## The Catalytic Mechanism for NleB/SseKs-Mediated Arginine GlcNAcylation

Several previous mutagenesis analyses showed that NleB, SseK1, SseK2, and SseK3 contain a putative catalytic DXD motif, and an exchange of these residues to alanine completely inhibited their glycosyltransferase activity (Li et al., 2013; Pearson et al., 2013; Wong Fok Lung et al., 2016). Moreover, present structural data indicated that all of the NleB, SseK1, SseK2, and SseK3 proteins share the GT-A fold with the conserved DXD motif (Esposito et al., 2018; Park et al., 2018; Ding et al., 2019; Newson et al., 2019). Typical for the GT-A family of the glycosyltransferase, the DXD motif in NleB and SseKs is involved in a divalent cation (Mn^2+^) coordination. Therefore, the NleB and SseK family proteins are GT-A type glycosyltransferase (Esposito et al., 2018; Park et al., 2018; Ding et al., 2019; Newson et al., 2019).

**Figure 3:**
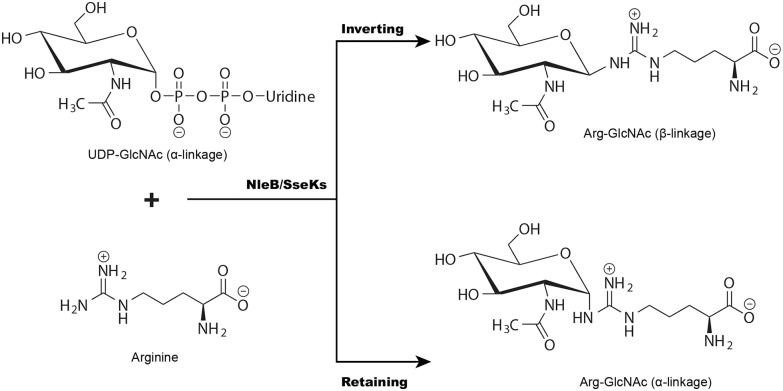
NleB/SseKs catalyze glycosyl group transfer with either inversion or retention of the anomeric stereochemistry. Glycosyltransferases (NleB/SseKs) can be segregated into “retaining” or “inverting” enzymes according to whether the stereochemistry of the glycosyl donor's anomeric bond is retained or inverted during the transfer.

Glycosyltransferases can be divided into “retaining” or “inverting” enzymes based on the stereochemistry of the glycosyl donor's anomeric bond during the glycosylation (Figure 3) (Lairson et al., 2008). Recently, the structures of GlcNAcylated TRADD-DD and RIPK1-DD were solved by one of our groups, revealing a β-configuration glycosidic linkage (Ding et al., 2019). Consistently, we previously reported the first synthesis of arginine GlcNAcylated peptides with a β-glycosidic linkage. Using these glycopeptides, we produced a monoclonal antibody that can specifically recognize GlcNAcylated TRADD-DD, FADD-DD, and RIPK1-DD (Pan et al., 2014). As the α-anomeric linkage in UDP-GlcNAc, these data strongly suggest that NleB is an inverting glycosyltransferase in catalyzing arginine GlcNAcylation (Figure 3). However, two retaining mechanisms have been proposed in the nuclear magnetic resonance (NMR) study of SseK1 and SseK3-catalyzed GlcNAc transfer (Esposito et al., 2018; Park et al., 2018). The major reasons for the discrepancy may due to the hydrolase activity of SseKs (Esposito et al., 2018). In the hydrolysis reaction, SseKs were function as hydrolases, a water molecule rather than the arginine acceptor executes the nucleophilic attack, thus generating an α-GlcNAc group (Esposito et al., 2018).

**Figure 4:**
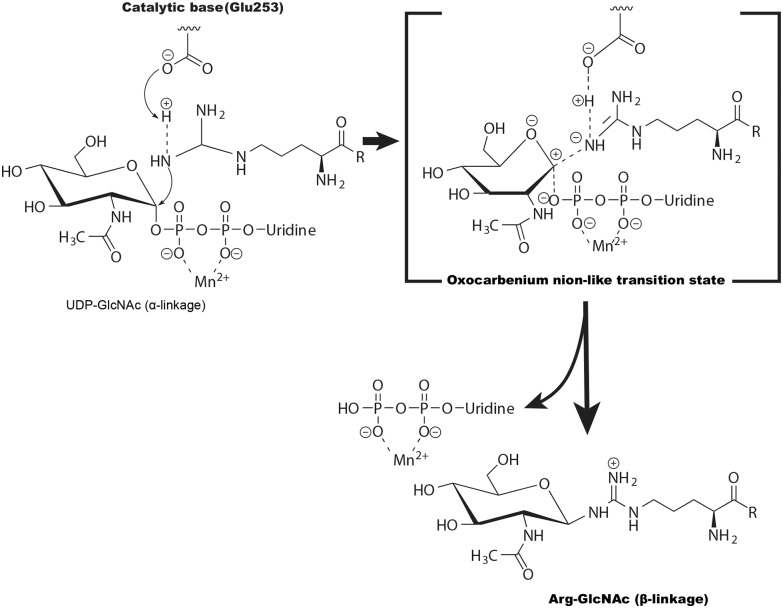
The potential catalytic mechanism (S_N_2-like) for NleB-catalyzed arginine GlcNAcylation. Glu253 of NleB acts as the base to deprotonate the guanidinium in TRADD (at Arg235), FADD (at Arg117), and RIPK1 (at Arg603). The arginine then nucleophilically attacks the C1 atom of UDP-GlcNAc, forming an oxocarbenium ion-like transition state that progresses to S_N_2 (substitution, nucleophilic, bimolecular)-like displacement of the UDP.

Glycosylation of arginine is unique because the guanidine group of arginine has a high acid dissociation constant value and is intrinsically an extremely poor nucleophile at physiological pH. The crystal structure of the NleB/FADD-DD complex showed that His182, His281, Tyr283, Tyr284, Trp329, and the negatively charged Glu253 might promote deprotonation of the arginine and therefore facilitate catalysis (Ding et al., 2019). Furthermore, the geometry of the UDP-GlcNAc and the position of the Arg117 in FADD-DD suggest NleB adopts a direct-displacement S_N_2 (substitution, nucleophilic, bimolecular)-like reaction to transfer the GlcNAc from the UDP-GlcNAc to the Arg117, and the Glu253 is proposed to act as the catalytic base (Figure 4) (Ding et al., 2019). Different from this, some other studies showed that SseK1, SseK2, and SseK3 may be retaining glycosyltransferases and adopt an S_N_i or orthogonal mechanism for the arginine GlcNAcylation reaction (Esposito et al., 2018; Park et al., 2018).

## The Catalytic Mechanism for EarP-Mediated Arginine Rhamnosylation

**Figure 5:**
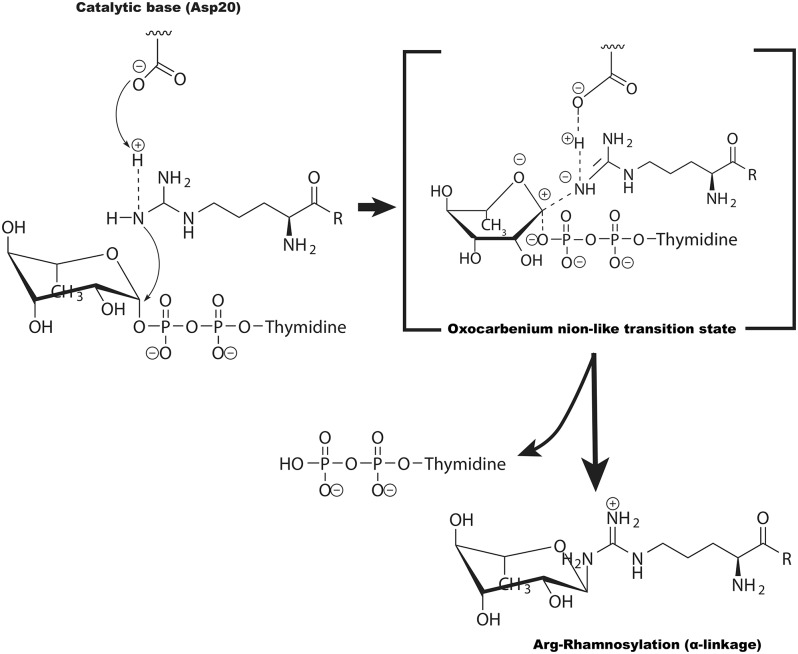
The potential catalytic mechanism (S_N_2-like) for EarP-catalyzed arginine GlcNAcylation. Asp20 of EarP acts as the base to deprotonate the guanidinium in EFP (at Arg32). The arginine then nucleophilically attacks the C1 atom of TDP-rhamnose, forming an oxocarbenium ion-like transition state that progresses to S_N_2-like displacement of the TDP.

Compared with the NleB family, the crystal structure of EarP, revealed a GT-B fold and acted as an inverting arginine rhamnosyltransferase (Krafczyk et al., 2017; Sengoku et al., 2018). In the arginine rhamnosylation reaction, the stereochemistry of the dTDP-β-L-rhamnose's anomeric bond is reversed, resulting in α-rhamnosyl on EF-P (Krafczyk et al., 2017; Wang et al., 2017; Sengoku et al., 2018). Upon successful inverting glycosyl transfer from dTDP-β-L-rhamnose to Arg32 in EF-P, the X-ray crystal structure of EarP/EF-P/dTDP-β-L-rhamnose complex together with the NMR data indicated that EarP probably performed an S_N_2 displacement reaction, with Asp20 as the general base (Figure 5) (Sengoku et al., 2018). Additionally, EarP-mediated arginine rhamnosylation requires the rhamnose ring of the dTDP-β-L-rhamnose to undergo a suitable structural change to expose the β-anomeric face of the rhamnose C1 atom (Sengoku et al., 2018).

## Conclusions

Arginine glycosylation, the attachment of sugar moieties (GlcNAc or rhamnose) to protein's arginine residue, is a novel type of posttranslational modification. There are three types of bacterial arginine glycosyltransferases that make this kind of modification known to date, including NleB homologs in pathogenic *E. coli* and *C. rodentium*, SseK homologs in *Salmonella*, and EarP family in *P. aeruginosa* and other relevant bacterial species. Interestingly, NleB/SseKs could also GlcNAcylate themselves, and self-GlcNAcylation of NleB, SseK1, and SseK3 is crucial for their biological activity during infection. Excitingly, the success in generating anti-Arg^GlcNAc^ and anti-Arg^Rha^ antibodies provides a powerful tool toward the discovery of novel arginine GlcNAcylated or rhamnosylated proteins, and this is the first step toward a comprehensive understanding of arginine glycosylation in nature (Pan et al., 2014; Li et al., 2016; Wang et al., 2017). Besides, a recent report provided evidence that 100066N and 102644N were acted as arginine-GlcNAc glycosyltransferase inhibitors (El Qaidi et al., 2018). Thus, these two compounds may have utility as reagents to further study arginine GlcNAcylation. In addition, the crystal structures of NleB, SseKs, EarP, arginine GlcNAcylated death domain-containing proteins, NleB/FADD-DD, and EarP/EF-P/dTDP-β-L-rhamnose provide compelling evidence of the catalytic mechanism for arginine glycosylation, which will be advantageous to us for designing NleB, SseKs, and EarP inhibitors against the certain pathogens.

## Author Contributions

XP, JL, and SL conceptualized the study. XP and JL prepared the original draft. All authors listed made a substantial intellectual contribution to the manuscript and edited and approved it for publication.

## Conflict of Interest

The authors declare that the research was conducted in the absence of any commercial or financial relationships that could be construed as a potential conflict of interest.
